# The “Hector” case and the community rehabilitation of offenders with Intellectual Disability and psychiatric disorders

**DOI:** 10.1192/j.eurpsy.2024.107

**Published:** 2024-08-27

**Authors:** J. Santambrogio, Emma Francia, Antonino Giancontieri, Massimo Clerici, Alessandro Santarone

**Affiliations:** ADELE BONOLIS AS.FRA. FOUNDATION, VEDANO AL LAMBRO, Italy

## Abstract

**Abstract:**

Hector, 44 years, with mild Intellectual Disability and impulse control disorder, committed serious sexual offences against two children of his partner. Considered “socially dangerous”, he was put in prison, then with a deferment of the enforcement of the prison sentence in the forms of home detention he was hospitalized in a psychiatric facility due to his depressive condition. Upon entering the Community, he presented a deflected mood as a reaction to the discomfort from the custodial experience, which was not cognitively integrated. Both psychotropic and rehabilitation treatments started. He has been involved in a gardening activity, too. After a first period of high degree of denial of the facts and a defensive mode marked by stolidity and fatuity, revealing his poor cognitive resources, during the psychiatric sessions he became even more conscious of his crime and the suffering of the victims. Services/pathways available for offenders with ID and psychiatric disorders will be presented.

**Disclosure of Interest:**

None Declared

